# Choroidal thickness in normal Indian subjects using Swept source optical coherence tomography

**DOI:** 10.1371/journal.pone.0197457

**Published:** 2018-05-16

**Authors:** Zeeshan Akhtar, Pukhraj Rishi, Ramasubramanian Srikanth, Ekta Rishi, Muna Bhende, Rajiv Raman

**Affiliations:** 1 Shri Bhagwan Mahavir Vitreoretinal Services, Sankara Nethralaya, Chennai, India; 2 Elite School of Optometry, Medical Research Foundation, Sankara Nethralaya, Chennai, India; 3 Srimathi Sundari Subramanian Department of Visual Psychophysics, Sankara Nethralaya, Chennai, India; 4 Sankara Nethralya-Orbis Paediatric Ophthalmology Learning & Training Centre, Sankara Nethralya, Tamil Nadu, India; Massachusetts Eye & Ear Infirmary, Harvard Medical School, UNITED STATES

## Abstract

**Aim:**

To determine choroidal thickness in healthy Indian subjects using Swept source optical coherence tomography (SS-OCT).

**Methods:**

In this prospective, observational, cross-sectional study; healthy Indian subjects (n = 230) with no history of ocular and/or systemic disorders were enrolled in the study. Choroidal thickness was measured for 230 eyes using SS-OCT. Subjects were divided into six age groups. Main outcome measures were subfoveal choroidal thickness (SFCT) and macular choroidal thickness (MCT) up to 3 mm at 500-micron interval from the fovea was measured in eight different quadrants.

**Results:**

The mean SFCT was 307±79 μm and mean MCT was 285±75 μm. No difference in the choroidal thickness was found among genders. Mean SFCT of the different age groups was 327±68 μm (12–18 years), 364±70 μm (18.1–30 years), 321±78 μm (30.1–40 years), 304±79 μm (40.1–50 years), 283±69 μm (50.1–60 years) and 262±72μm (above 60 years; p <0.001; One way ANOVA). Mean macular choroidal thickness was 305±60 μm, 342±61 μm, 306±72 μm, 282±79 μm, 261±66 μm, 238±68μm respectively (p<0.001; one way ANOVA). A significant weak negative correlation was found between age with SFCT (r = -0.368, p<0.001) and MCT (r = -0.40, p<0.001). No significant correlation was found with refractive error, axial length and ocular perfusion pressure.

**Conclusion:**

This study showed that mean SFCT and MCT was 307±79μm and 285±75 μm, respectively, among healthy Indian subjects. Mean CT was found to decrease with age, although there was no difference in CT between genders.

## Introduction

The choroid, with metabolic support of retinal pigment epithelium (RPE), provides nourishment and blood supply to the outer retina, and pre-laminar portion of the optic nerve.[1,2] Abnormalities of choroid have been a major concern in the pathophysiology of chorio-retinal diseases [3,4] such as central serous chorioretinopathy (CSC), [5,6] Vogt-Koyanagi-Harada (VKH) disease, [7] polypoidal choroidal vasculopathy (PCV), age-related macular degeneration (AMD), [8,9] high myopia, [10] and diabetes mellitus (DM). [11] An advanced, non-invasive and quick method used in structural analysis of choroid is Optical coherence tomography (OCT) which works on the principle of low coherence interferometry.[12] Spaide et al, introduced a technique of “Enhanced Depth Imaging” (EDI) using spectral domain (SD) OCT by capturing inverted images in which choroid is placed near zero delay, providing clear visualization of the choroidal structure.[13] However in EDI OCT, improved visualization of choroid is achieved by compromising resolution of retinal layers.

Swept source (SS) OCT has the potential of simultaneous imaging of the retina and choroid without EDI. It uses high penetration OCT probe with wavelength of 1050 nm and improved resolution compared to SD-OCT.[14] SS-OCT can reproducibly measure Choroidal thickness (CT) in 100% of eyes with an acquisition rate of 100,000 A scans/sec whereas SD-OCT can measure the same, only in 74.4% of eyes at a rate of 27,000 A scans/sec.[15] As the reproducibility and penetration is higher in SS-OCT, it can provide more accurate measurement of choroidal thickness compared to SD-OCT.

There have been a few studies reporting choroidal thickness in normal subjects.[16–19] However, contradictory results have become known through literature about the influence of ocular and systemic parameters on choroidal thickness. This study provides normative data for choroidal thickness in healthy Indian subjects of different age groups using SS-OCT, and its relationship to other ocular and systemic parameters.

## Materials and methods

Ethics subcommittee of Vision Research Foundation granted the permission to conduct this study. This study led to the award of M.Phil (optometry) for the first author. The study was approved by Institutional Review Board (IRB) and followed the principles of Declaration of Helsinki. However, IRB did not approve of contact procedures (corneal thickness, Ap-planation tonometry) in subjects younger than 18 years of age, and these procedures were not performed on this group of participants. A duly signed informed consent was obtained from all the participants after explaining the nature and possible consequences of the study. Written informed consent was obtained from parents or guardians of the participants under age 18 included in the study. A prospective, observational, cross-sectional study was performed between July 2015 and September 2016. Healthy Indian subjects (n = 230) aged 12–80 years with no history of ocular and/or systemic disorders were enrolled in the study. All subjects had best corrected visual acuity (BCVA) of 6/9 or better in one or both eyes. Exclusion criteria included subjects with refractive error >±6 D, any abnormality detected during OCT scan, poor image quality due to dense cataract or unstable fixation, and any history of ocular surgery in the last 3 months. Sample size (n = 229) was calculated using specified precision method and 95% confidence interval.

All participants underwent comprehensive ophthalmic evaluation including visual acuity testing using Snellen’s acuity chart, slit lamp biomicroscopy, intraocular pressure (IOP) using Goldmann applanation tonometer, and dilated fundus evaluation using indirect ophthalmoscope. Central corneal thickness (CCT) was measured using DGH ultrasonic Pachymeter Pachette 2® (DGH Technology Inc., 110 Summit Drive, Suite B, Exton, PA 19341, USA), axial length (AXL) measurement was performed using ultrasound biometry (OcuScan® RxP, Alcon Laboratories, 6201 South Freeway, Fort Worth, TX, USA).

### Blood pressure measurement

Blood pressure (BP) was recorded in the right arm in sitting position after 5 minutes of rest using a mercury sphygmomanometer (Diamond® Industries, Pune, India). Two readings were recorded to ensure stable hemodynamics and average readings were used for analysis. Ocular Perfusion Pressure: Ocular perfusion pressure (OPP) was calculated using the following formula; Mean ocular perfusion pressure (OPP) = 2/3mean arterial pressure (MAP)–IOP, where MAP = diastolic BP + 1/3 (Systolic BP–Diastolic BP). Systolic and diastolic OPP were calculated using the following equation: Systolic OPP = systolic BP—IOP. IOP used in the formula was adjusted for CCT using Ehler’s formula.

### Choroidal thickness

**Fig 1:**
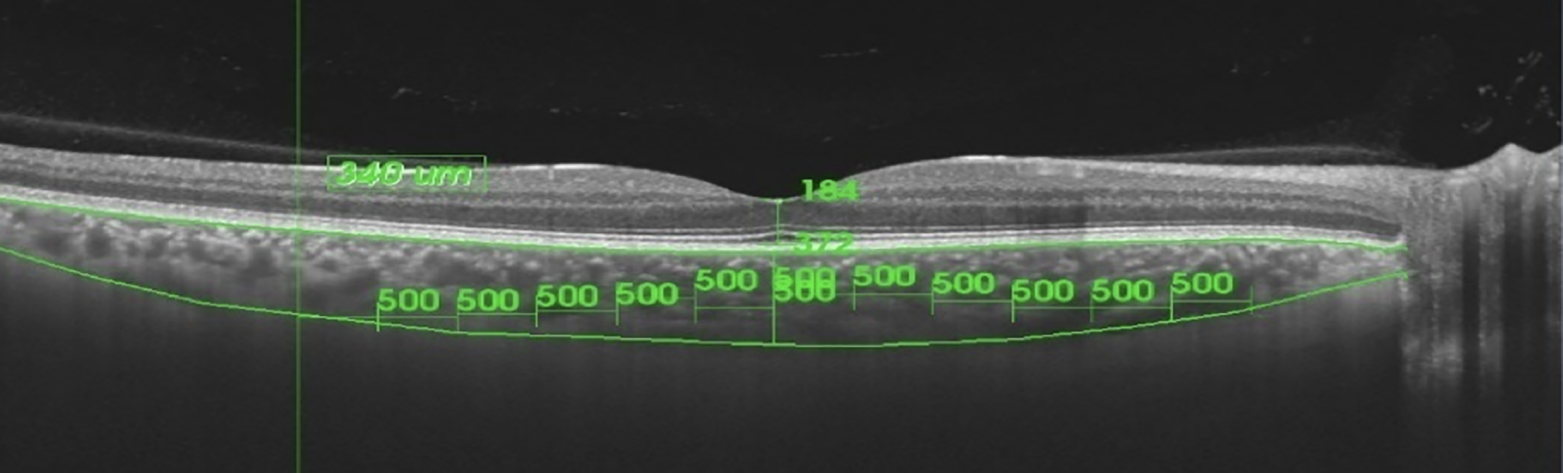
OCT scan showing choroidal thickness measurement at 13 different locations.

OCT scans were obtained using SS-OCT (Deep Range Imaging, OCT -1, Atlantis, Topcon, Tokyo, Japan). Scan used for imaging is a 12-mm horizontal, vertical and radial scan (x2) centred on fovea. Measurements were taken at fovea and at 500 microns interval nasal, temporal, superior, inferior, infero-nasal, supero-temporal, infero-temporal and supero-nasal up to 3000 microns from fovea (Fig 1). Choroidal thickness was measured from the base of hyper-reflective RPE-Bruch’s membrane to the choroid-scleral interface. Six measurements were taken for each quadrant and average values were used for analysis. For each eye, choroidal thickness was measured at 52 different points. A mean choroidal thickness for each quadrant (Temporal, Nasal, Superior, Inferior, Supero-nasal, Infero-temporal, Supero-temporal and Infero-nasal) was obtained by calculating the average value of 6 points for each quadrant. All measurements were manually recorded. Variability in the manual measurement was assessed for 15 random eyes using a horizontal line scan. Measurements were repeated by two masked observers for 13 different locations including subfoveal and 6 different locations nasal and temporal to fovea. Inter observer variability was assessed by comparing the values between two observers. Repeatability of the manual measurements was assessed by repeating the measurements twice at 13 different locations by the same observer using the horizontal line scan with minimum time gap of one week. All examination procedures were conducted between 11:00 am to 3:00 pm to control for diurnal variation. The statistical tests used in the study are listed in S1 Table.

## Results

A total of 236 subjects were screened for the study, of which two hundred and thirty eyes of 230 healthy subjects (125 male & 105 female subjects) were included. Six subjects were excluded due to poor image quality (n = 3), glaucoma suspect (n = 1), and retinal pathologies (n = 2). The median age was 44.0±29.0 years, ranging between 12 and 80 years. Details of the ocular and systemic parameters are shown in Tables 1 and 2, respectively.

**Table 1:** Choroidal thickness in normal Indian subjects using SS-OCT: Details of ocular parameters in different age groups.

Age Groups (years)	RE (Diopter) (Mean±SD) (n = 230)	IOP (mm Hg) (Mean±SD) (n = 230)	C_IOP (mm Hg) (Mean±SD) (n = 200)	AXL (mm) (Mean±SD) (n = 200)	CCT (μm) (Mean±SD) (n = 200)
Group 1 (12.1–18) (n = 30)	-0.12±1.81*	12.70±1.68	—	—	—
Group 2 (18.1–30) (n = 35)	-0.25±2.25*	12.71±2.09	12.10±2.59	23.27±0.86	528.5±34.7
Group 3 (30.1–40) (n = 33)	-0.12±1.18*	13.45±1.93	12.63±2.74	23.20±1.14	531.5±35.4
Group 4 (40.1–50) (n = 41)	0.00±0.31*	13.39±2.13	12.80±3.10	23.00±0.68	528.2±33.6
Group5 (50.1–60) (n = 51)	0.87±2.12*	13.0±3.0*	13.3±3.0	22.9±0.76	521.1±35.3
Group 6 (>60) (n = 40)	0.00±1.75	14.0±3.0*	13.6±3.3	22.8±0.62	519.0±33.1
*p* value	<0.001†	0.2†	0.1‡	0.04‡	0.4‡
Total (n = 230)	0.00±1.25*	13.0±3.0*	12.9±3.0	22.98±0.82	525.0±31.4

RE = Refractive error, IOP = Intra ocular pressure, AXL = Axial Length, C_IOP = IOP corrected for CCT, CCT = central corneal thickness.

*Median±IQR

†Kruskal Wallis

‡ ANOVA

**Table 2:** Choroidal thickness in normal Indian subjects using SS-OCT: Details of systemic parameters in different age groups.

Age Groups	SYS_BP (n = 230)	DIA_BP (n = 230)	MAP (n = 230)	OPP (n = 230)	C_OPP (n = 200)
Group 1	110.0±20.0*	70.0±13.0*	83.33±16.6*	42.18±5.49	—
Group 2	110.0±10.0*	70.0±10.0*	86.66±10.0*	45.75±5.80	46.36±5.85
Group 3	120.0±20.0*	80.0±10.0*	91.81±8.25	47.75±6.00	48.58±6.27
Group 4	120.0±20.0*	80.0±10.0*	91.32±7.91*	47.49±5.37	48.07±5.74
Group 5	130.0±20.0*	80.0±10.0*	95.06±7.62	49.92±4.83	50.0±5.52
Group 6	130.0±20.0*	80.0±10.0*	96.66±6.66*	50.22±5.49	50.15±5.63
*p* value	<0.001†	<0.001†	<0.001†	<0.001†	0.02†
TOTAL	120.0±20.0*	80.0±10.0*	93.33±13.3*	47.58±5.99	48.76±5.88

SYS_BP = systolic blood pressure, DIA_BP = Diastolic blood pressure, MAP = Mean arterial pressure, OPP = ocular perfusion pressure, C_OPP = ocular perfusion pressure calculated using CCT corrected.

* Median ± IQR

†Kruskal Wallis test

‡ One Way ANOVA.

Systolic and Diastolic Blood pressure, and mean arterial pressure was significantly different between the six groups (Kruskal Wallis test, p <0.001). Ocular perfusion pressure (C_OPP) was also different between the five groups (One way ANOVA 0.02). Group 1 showed a significantly lower systolic and diastolic blood pressure, mean arterial pressure and ocular perfusion pressure than groups 3, 4, 5 and 6 (Post hoc Mann-Whitney, p <0.008). Groups 5 and 6 had higher systolic blood pressure and mean arterial pressure than group 2. It also showed significantly lower systolic BP than Group 3 & 4. Group 3 revealed lower systolic BP when compared to Groups 5 and 6. Group 6 was found to have higher systolic BP and mean arterial pressure (MAP) than group 4 (post hoc Mann Whitney <0.008). The choroidal thickness among different age groups for all quadrants including SFCT and MCT is represented (across rows) in Table 3 (ANOVA p value <0.05).

**Table 3:** Choroidal thickness in normal Indian subjects using SS-OCT: Mean choroidal thickness (microns) in different quadrants amongst various age groups.

Fundus Quadrant (Mean±SD)	Group 1 (n = 30)	Group 2 (n = 35)	Group 3 (n = 33)	Group 4 (n = 41)	Group 5 (n = 51)	Group 6 (n = 40)	*p* value^§^	Mean (n = 230)
SFCT	327±68	364±70	321±78	304±79	283±69	262±72	*<0*.*001*	307±79
Temporal	316±69	349±80	299±85	284±79	260±63	238±63	*<0*.*001*	287±81
Nasal	265±75	295±59	277±78	260±79	242±72	216±88	*0*.*003*	257±79
Superior	332±62	382±82	331±71	312±82	288±73	270±89	*<0*.*001*	315±84
Inferior	311±60	350±67	320±82	279±87	261±68	232±76	*<0*.*001*	288±82
IT	305±57	337±68	294±73	268±84	243±68	216±66	*<0*.*001*	272±80
SN	298±65	340±72	306±76	288±85	269±70	250±82	*0*.*001*	289±80
ST	323±63	355±67	316±76	295±86	274±75	259±103	*<0*.*001*	300±86
IN	288±67	328±64	302±81	274±89	250±75	221±79	*<0*.*001*	273±83
*p* value^#^	*0*.*003*	*<0*.*001*	0.2	0.1	0.007	0.01		*<0*.*001*
MCT	305±60	342±61	306±72	282±79	261±66	238±68	*<0*.*001*	285±75

SFCT = Subfoveal choroidal thickness, MCT = Macular choroidal thickness, IT = Infero-temporal, SN = Supero-nasal, ST = Supero-temporal, IN = Infero-nasal.

p value §# (across rows in blue) shows difference among the groups. p value # (across columns in green) shows difference between the quadrants for different age groups.

§ One way ANOVA (Bonferroni p <0.01).

# One way ANOVA (Bonferroni p <0.005)

**Fig 2:**
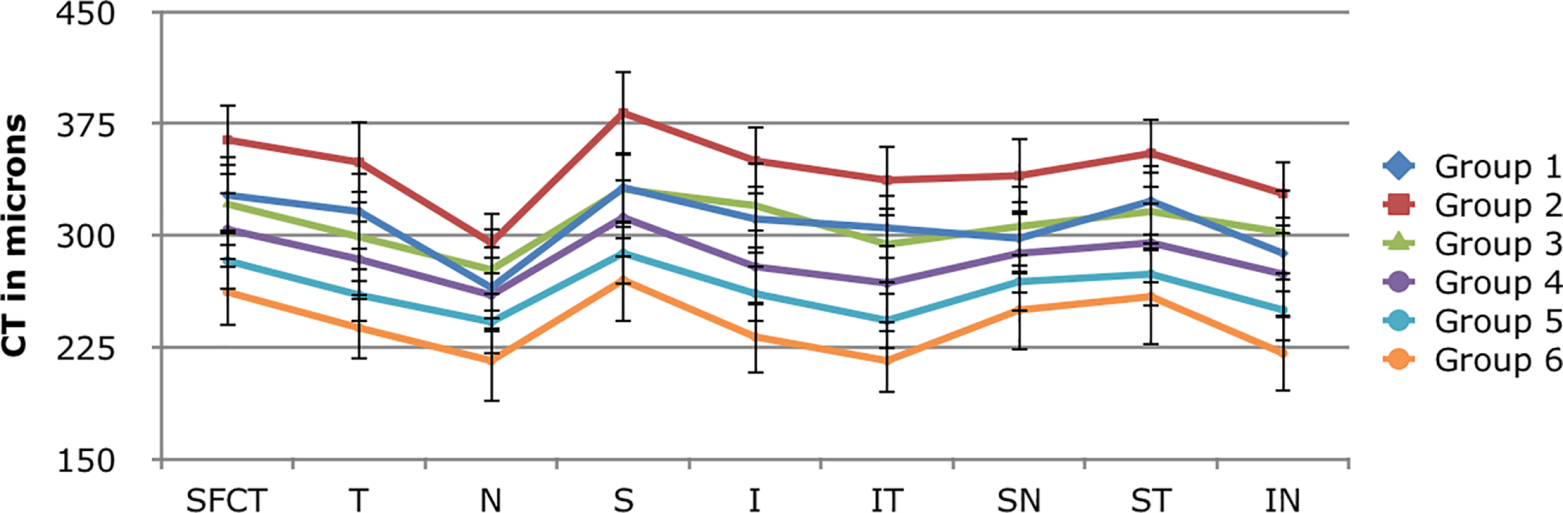
Graph showing choroidal thickness profile with mean and confidence interval among different age groups.

Groups 5 and 6 showed a significantly lower CT in all the quadrants including SFCT and MCT when compared to Group 2; Group 6 shows lower CT in temporal, inferior, infero-temporal, infero-nasal, MCT and SFCT than group 1. Group 3 was found to have thicker choroid in MCT, temporal, inferior, infero-nasal and infero-temporal quadrants than Group 6. Group 4 revealed lower choroidal thicknesses in superior, inferior, temporal and infero-temporal quadrants including SFCT & MCT when compared to Group 2 (conservative p value <0.008). The difference between the quadrants for different age groups is represented (across columns) in Table 3. CT was found to be thickest in superior quadrant and thinnest in nasal quadrant. Significant difference was observed between quadrants in only group 1 and 2 (one way ANOVA p value <0.05). CT in nasal quadrant was lesser than superior quadrant in group 1 and 2 (Post Hoc p value <0.005) whereas no difference was noted between other quadrants. Intra-observer reproducibility and inter-observer repeatability was greater than 0.9 at all locations. There was a gradual decrease in choroidal thickness with increasing age in all quadrants. The trend of choroidal thickness with mean and confidence interval in different age group is shown in Fig 2.

**Fig 3:**
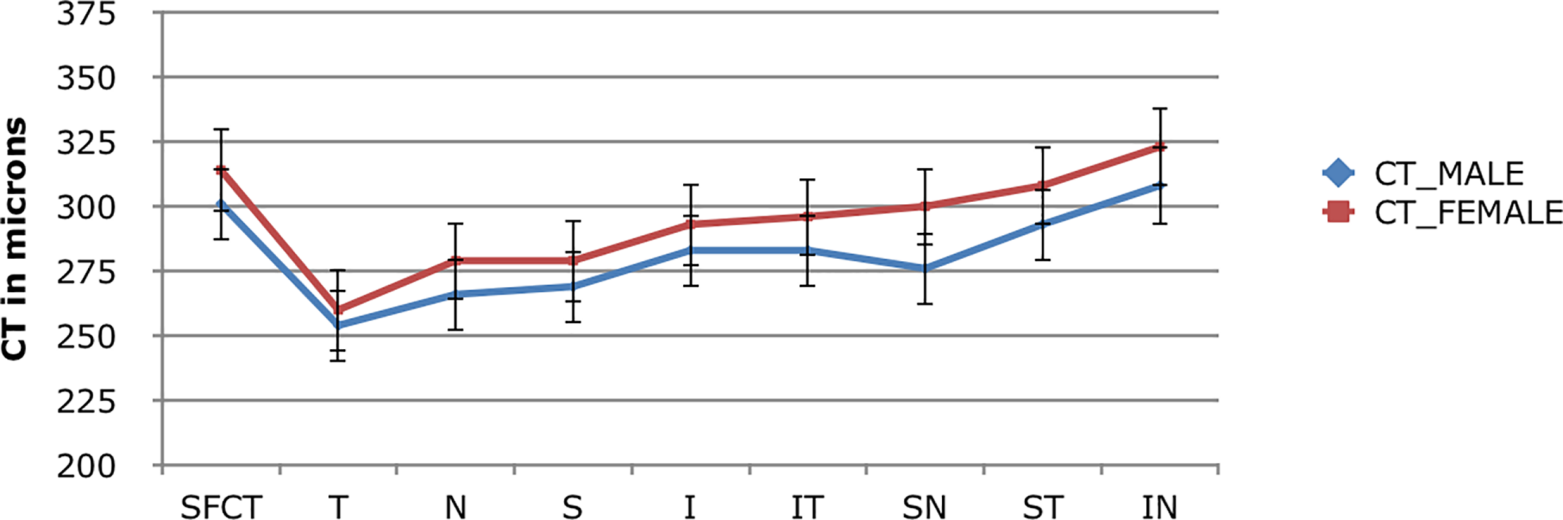
Graph with mean choroidal thickness with confidence interval amongst male and female subjects.

Choroidal thickness was compared between males and females in all quadrants. The SFCT was 301.10 ± 75.25 microns in males and 314.01 ± 83.74 microns in females whereas MCT was 279.50 ± 74.02 microns and 292.83 ± 77.67 microns in males and females respectively (Independent t test, p> 0.05). No statistically significant difference in CT was found between males compared to females in any of the quadrants, although females were found to have slightly thicker choroid then males in all the quadrants (Fig 3).

**Fig 4:**
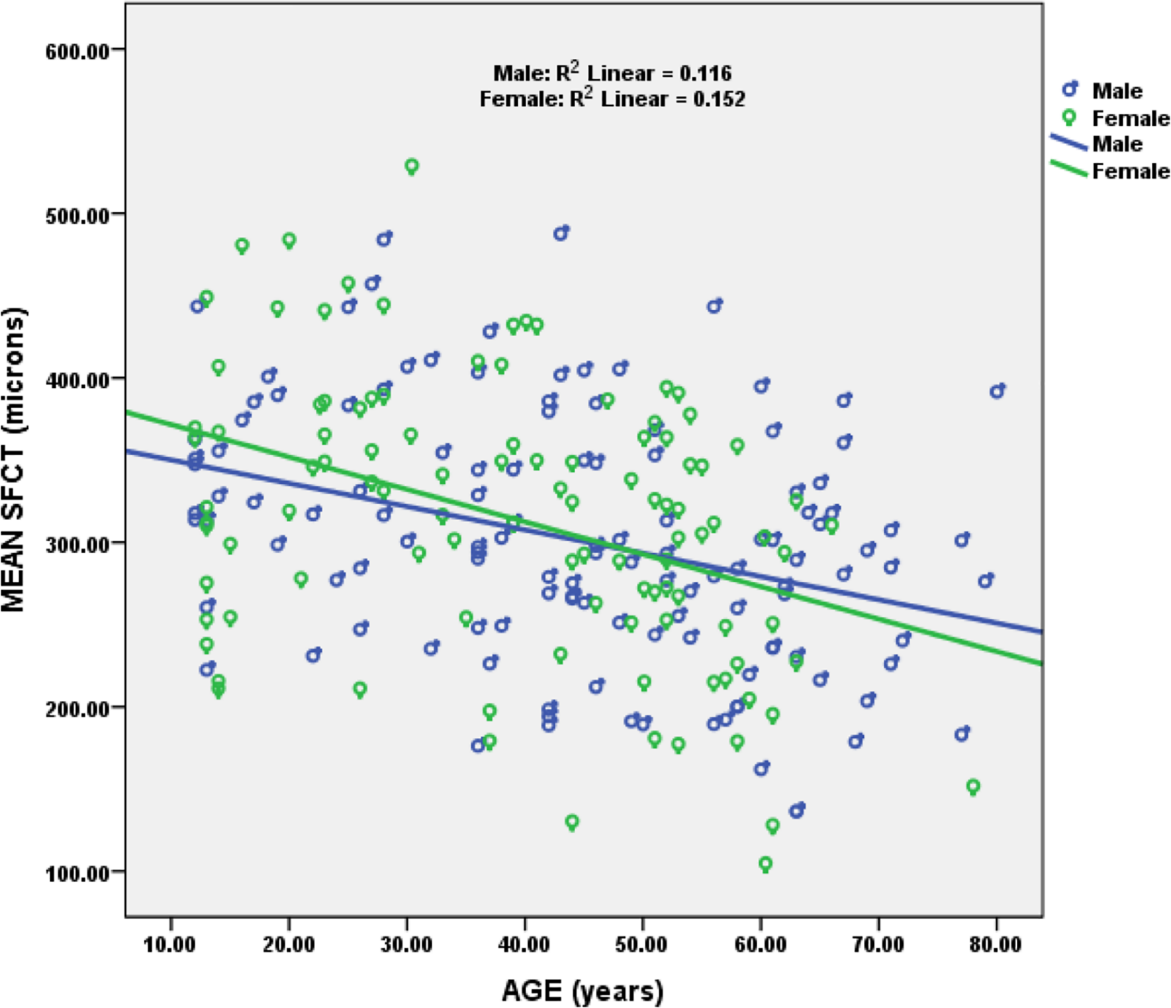
Scatter plot showing negative correlation between SFCT and age.

**Fig 5:**
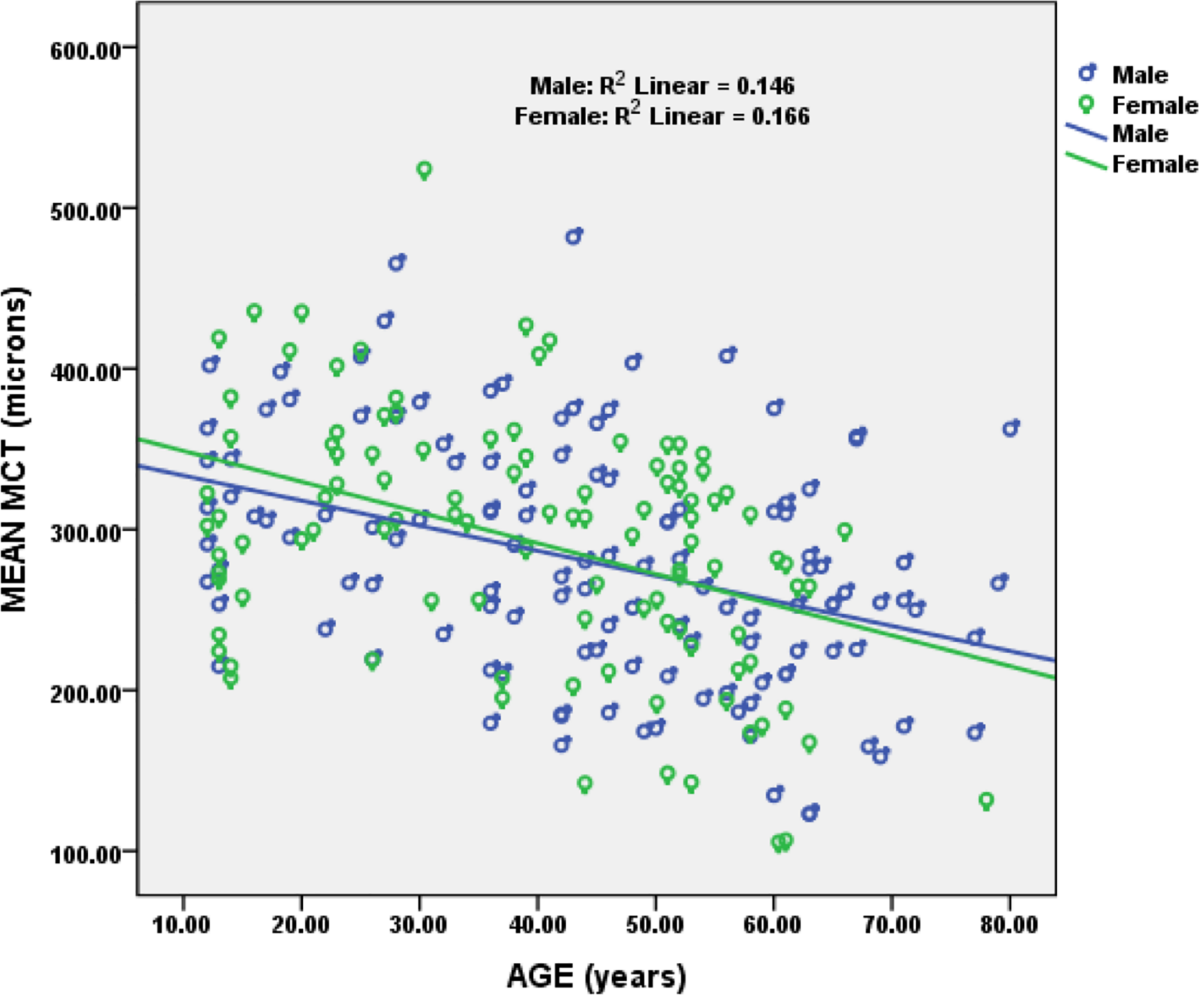
Scatter plot showing negative correlation between MCT and age.

The relationship between age with SFCT and macular choroidal thickness (MCT) was assessed using Spearman’s correlation. We found a significant negative correlation between age with SFCT (r = -0.368, p = <0.001, Fig 4) & MCT (r = -0.40, p = <0.001, Fig 5) whereas no significant correlation was found with other parameters such as refractive error, axial length and ocular perfusion pressure. SFCT and MCT multiple regression analysis including age, refractive error, axial length and ocular perfusion pressure was performed. The equation predicted SFCT is as follows:

(SFCT=‑2.496(age)+6.095(RE)‑3.882(AXL)‑0.245(OPP)+521.745,R2=0.194,p=<0.001)

The equation predicted MCT is as follows:

(MCT=‑2.665(age)+6.968(RE)‑5.039(AXL)‑0.195(OPP)+532.570,R2=0.238,p=<0.001)

**Fig 6:**
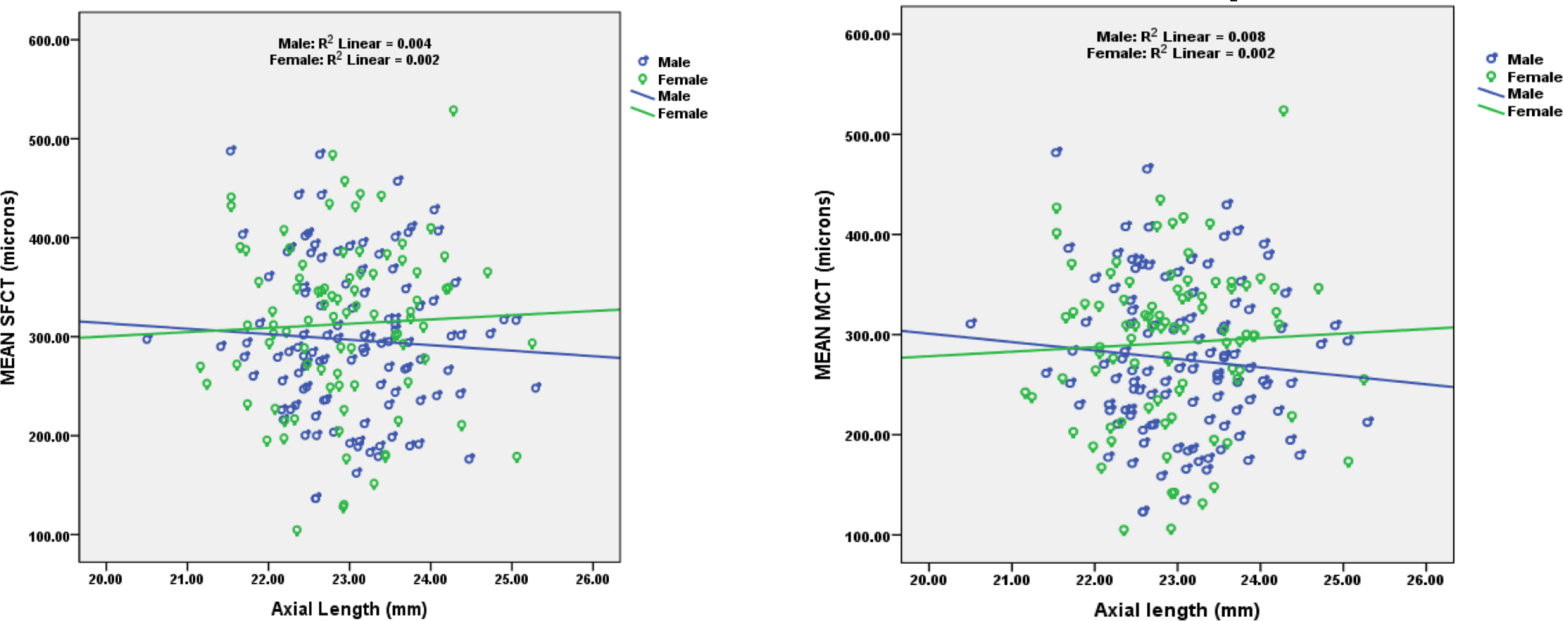
Scatter plot showing correlation between axial length, and subfoveal choroidal thickness (left) and mean choroidal thickness (right).

Relationship of axial length (20.50 to 25.29 mm) with choroidal thickness (SFCT and MCT) was assessed using Pearson correlation (Fig 6). A weak negative correlation was found between axial length and choroidal thickness (SFCT & MCT) which was not statistically significant. Pearson SFCT (r = -0.018, n = 200 p = 0.80), MCT (r = -0.034, n = 200, p = 0.63).

**Fig 7:**
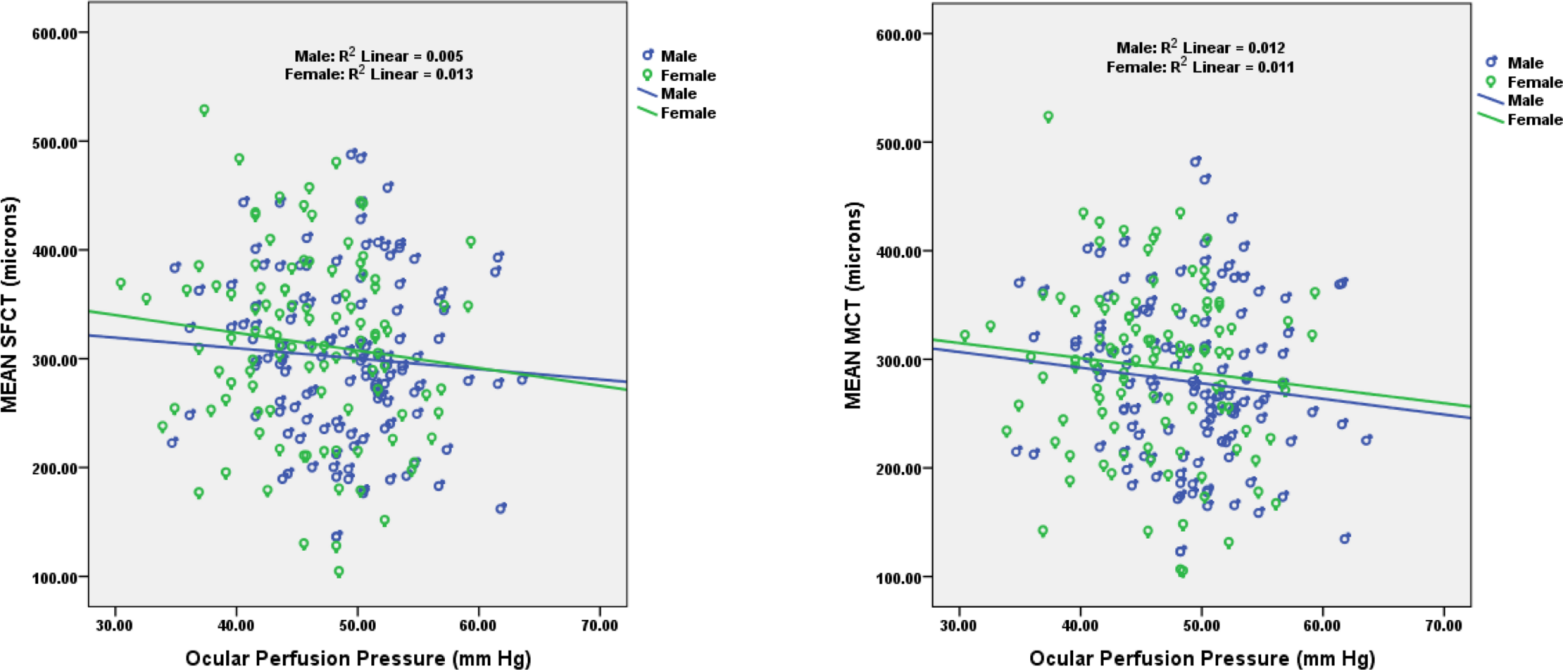
Scatter plot showing the correlation between ocular perfusion pressure and SFCT (left) and MCT (right).

**Fig 8:**
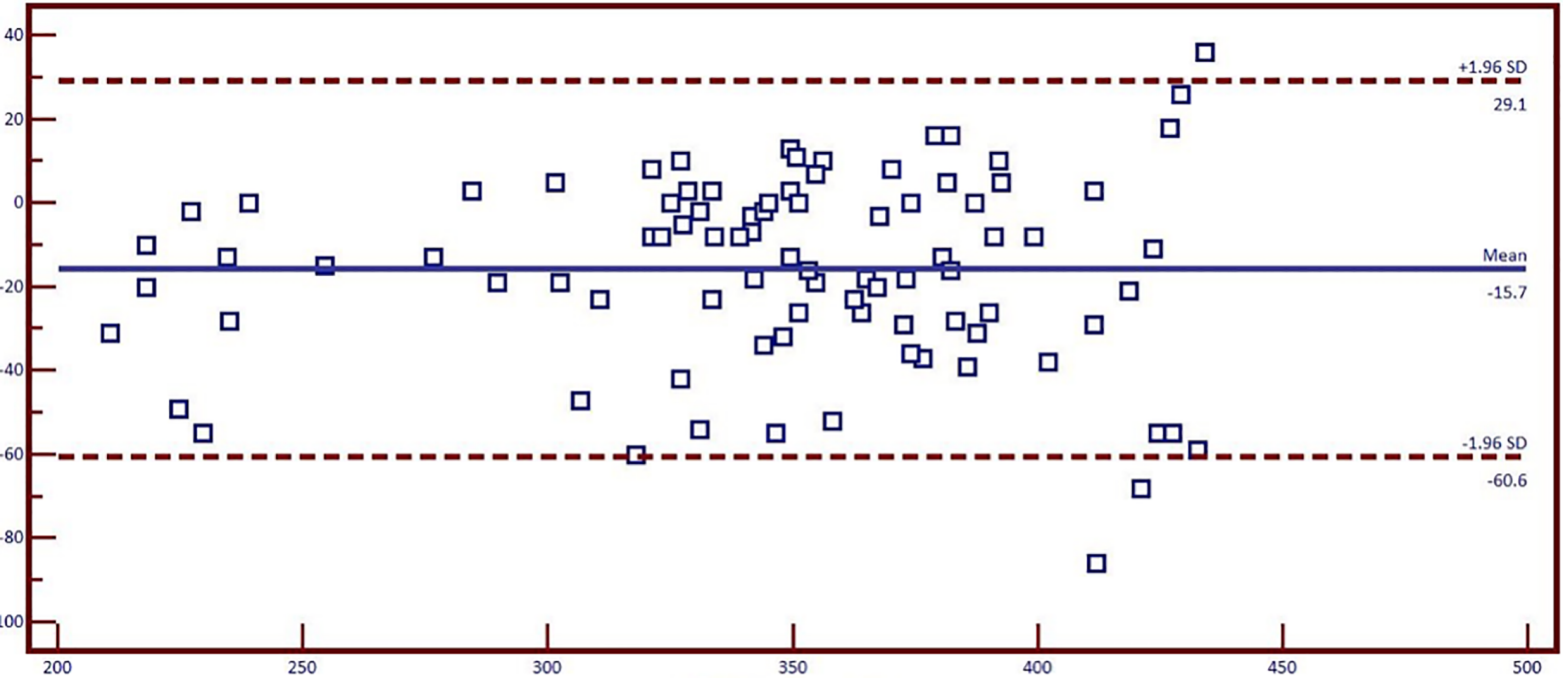
Bald-Altman plot showing inter-observer variability between two observers.

Correlation between Ocular perfusion pressure (OPP) and choroidal thickness was assessed using Pearson correlation. Though not statistically significant, poor negative correlation was noted between OPP with SFCT (r = -0.123, n = 200, p = 0.08), borderline correlation was found between MCT and OPP (r = -0.130, n = 200, p = 0.066, Fig 7). Table 4 gives the repeatability measures of the manual measurements of the choroidal thickness. Intra-class correlation coefficients (ICC) of choroidal thickness measured at different points are given in the table. It was found that choroidal thickness measurement at different points was highly repeatable with high ICC values. Inter-observer variability: Agreement between two observers in manual measurement of choroidal thickness was assessed for 15 eyes using Bland Altman Plot (Fig 8). A good agreement was found between two observers for 13 different point locations. Table 5 shows the mean difference with 95% CI (confidence interval) for 13 different points between two observes.

**Table 4:** Repeatability measures of choroidal thickness at different point locations.

Point of Measurement	ICC (95% CI)
TEMPORAL 6 (T6)	0.999 (0.996–1.00)
TEMPORAL 5 (T5)	0.999 (0.998–1.00)
TEMPORAL 4 (T4)	0.999 (0.997–1.00)
TEMPORAL 3 (T3)	0.999 (0.997–1.00)
TEMPORAL 2 (T2)	0.999 (0.997–1.00)
TEMPORAL 1 (T1)	0.998 (0.994–0.999)
SFCT	0.990 (0.969–0.997)
NASAL 1 (N1)	0.999 (0.997–1.00)
NASAL 2 (N2)	0.914 (0.744–0.971)
NASAL 3 (N3)	0.999 (0.997–1.00)
NASAL 4 (N4)	0.999 (0.998–1.00)
NASAL 5 (N5)	1.00 (0.999–1.00)
NASAL 6 (N6)	0.999 (0.998–1.00)

**Table 5:** Mean difference between the two observers for CT measurements.

Point of measurement	Mean Difference (95% CI)
TEMPORAL 6 (T6)	-17.6 (28.6 to -63.8)
TEMPORAL 5 (T5)	-22.1 (34.0 to -78.3)
TEMPORAL 4 (T4)	-17.6 (31.7 to -66.9)
TEMPORAL 3 (T3)	-14.7 (29.9 to -59.4)
TEMPORAL 2 (T2)	-12.5 (31.1 to -56.0)
TEMPORAL 1 (T1)	-9.9 (18.5 to -38.2)
SFCT	-1.00 (15.4 to -17.4)
NASAL 1 (N1)	-3.6 (25.7 to -32.9)
NASAL 2 (N2)	-6.1 (31.4 to -43.6)
NASAL 3 (N3)	-11.1 (22.7 to -45.0)
NASAL 4 (N4)	-5.1 (23.6 to -33.9)
NASAL 5 (N5)	-7.1 (58.6 to -72.7)
NASAL 6 (N6)	3.1 (41.7 to -35.6)

## Discussion

To the best of our knowledge, there is no previously published study across all age groups including subjects below 18 years in Indian ethnicity that include all possible factors affecting choroidal thickness. The choroidal thickness reported in our study are comparable to those reported by Agawa et al and Ikuno et al using SS OCT. [16,20] They have found choroidal thickness to be thickest in superior quadrant followed by inferior, temporal and nasal quadrants with a similar trend as we found in current study. The values in our study were approximately 50 microns thinner than those reported by Agawa et al and Ikuno et al. However, the differences in the choroidal thickness may result due to diurnal variation and difference in the subject’s profile such as age, axial length and refractive error.

In our study of healthy subjects, age has a significant negative association with choroidal thickness. The association between choroidal thickness and age was also reported by Wen Bin et al and Ding et al, they found a significant decline in choroidal thickness by 3.3 microns and 5.4 microns respectively per year of age. [19,21] Ding et al has found a significant decrease in CT only in subjects above 60 years of age. However, in our study we found a 1.66 microns decrease in SFCT per year of age which was comparable to 1.18 microns in a study by Chhablani et al in Indian subjects. [17] It has been reported that choroidal thickness decreases with increasing axial length. [14,19] However, this was more prominent in subjects with axial length >25 mm. Studies performed on axial length less than 25 mm did not report any effect of axial length on choroidal thickness. [16,17] However, in current study we did not find any significant relationship between SFCT & MCT with axial length. This finding can be attributed to the inclusion of subjects with axial length ranging between 20.50 mm and 25.29 mm to assess the normal variation of choroidal thickness in healthy subjects. Studies have reported that choroidal thickness increases with increase in refractive error (<±6 Diopters). [16,22] Whereas few studies have reported a negative correlation between CT and Refractive error. [18,19] Current study did not find any relationship between refractive error and CT as majority of participants were emmetropic or mild myopic or hyperopic.

Considering the vascular nature of choroid, OPP can influence the normal CT. Rishi et al reported that high MOPP (mean ocular perfusion pressure) could be one of the etiologic mechanisms for structural change in choroid. [8] However, in current study no significant correlation was found between ocular perfusion pressure and choroidal thickness.

The normal choroidal thickness has also been reported in younger population (below 18 years) using SS OCT. In the present study, the mean SFCT and MCT were 327 and 305 microns respectively. Our finding of SFCT was approximately 10 to 15 microns greater than reported by other studies using SS OCT in subjects below 18 years. [23,24] We found the choroidal thickness was significantly different between teenagers’ and elderly aged group (>60 years). These results were found to be similar as findings of Ruiz Mareno et al using SS OCT. According to our results, the choroidal thickness decreases with increasing age, predominantly with subjects above 50 years of age. No difference was found between genders.

## Supporting information

S1 Table Statistical tests used in the study. (DOCX)

## References

[1] Nickla DL, Wallman J. The multifunctional choroid. Prog Retin Eye Res. 2010;29: 144–168. doi: 10.1016/j.preteyeres.2009.12.002 20044062 10.1016/j.preteyeres.2009.12.002 PMC2913695

[2] Kim M, Kim SS, Kwon HJ, Koh HJ, Lee SC. Association between choroidal thickness and ocular perfusion pressure in young, healthy subjects: enhanced depth imaging optical coherence tomography study. Invest Ophthalmol Vis Sci. 2012;53: 7710–7717. doi: 10.1167/iovs.12-10464 23092924 10.1167/iovs.12-10464

[3] Spaide RF. Age-Related Choroidal Atrophy. Am J Ophthalmol. 2009;147: 801–810. doi: 10.1016/j.ajo.2008.12.010 19232561 10.1016/j.ajo.2008.12.010

[4] Imamura Y, Fujiwara T, Margolis R, Spaide RF. Enhanced depth imaging optical coherence tomography of the choroid in central serous chorioretinopathy. Retina 2009;29: 1469–1473. doi: 10.1097/IAE.0b013e3181be0a83 19898183 10.1097/IAE.0b013e3181be0a83

[5] Maruko I, Iida T, Sugano Y, Ojima A, Ogasawara M, Spaide RF. Subfoveal choroidal thickness after treatment of central serous chorioretinopathy. Ophthalmology 2010;117: 1792–1799. doi: 10.1016/j.ophtha.2010.01.023 20472289 10.1016/j.ophtha.2010.01.023

[6] Hamzah F, Shinojima A, Mori R, Yuzawa M. Choroidal thickness measurement by enhanced depth imaging and swept-source optical coherence tomography in central serous chorioretinopathy. BMC Ophthalmol. 2014;14: 145 doi: 10.1186/1471-2415-14-145 25421855 10.1186/1471-2415-14-145 PMC4255445

[7] Fong AHC, Li KKW, Wong D. Choroidal evaluation using enhanced depth imaging spectral-domain optical coherence tomography in Vogt-Koyanagi-Harada disease. Retina 2011;31:.502–509. doi: 10.1097/IAE.0b013e3182083beb 21336069 10.1097/IAE.0b013e3182083beb

[8] Rishi P, Rishi E, Mathur G, Raval V. Ocular perfusion pressure and choroidal thickness in eyes with polypoidal choroidal vasculopathy, wet-age-related macular degeneration, and normals. Eye 2013;27: 1038–1043. doi: 10.1038/eye.2013.106 23764988 10.1038/eye.2013.106 PMC3772351

[9] Chung SE, Kang SW, Lee JH, Kim YT. Choroidal thickness in polypoidal choroidal vasculopathy and exudative age-related macular degeneration. Ophthalmology 2011;118:840–845. doi: 10.1016/j.ophtha.2010.09.012 21211846 10.1016/j.ophtha.2010.09.012

[10] Ho M, Liu DTL, Chan VC, Lam DS. Choroidal thickness measurement in myopic eyes by enhanced depth optical coherence tomography. Ophthalmology 2013;120: 1909–1914. doi: 10.1016/j.ophtha.2013.02.005 23683921 10.1016/j.ophtha.2013.02.005

[11] Esmaeelpour M, Brunner S, Ansari-Shahrezaei S, Nemetz S, Povazay B, Kajic V, et al Choroidal thinning in diabetes type 1 detected by 3-dimensional 1060 nm optical coherence tomography. Invest Ophthalmol Vis Sci. 2012;53: 6803–6809. doi: 10.1167/iovs.12-10314 22952126 10.1167/iovs.12-10314

[12] Gabriele ML, Wollstein G, Ishikawa H, Kageman L, Xu J, Folio LS, et al Optical coherence tomography: history, current status, and laboratory work. Invest Ophthalmol Vis Sci. 2011;52: 2425–2436. doi: 10.1167/iovs.10-6312 21493951 10.1167/iovs.10-6312 PMC3088542

[13] Spaide RF, Koizumi H, Pozzoni MC. Enhanced depth imaging spectral-domain optical coherence tomography. Am J Ophthalmol. 2008;146:.496–500. doi: 10.1016/j.ajo.2008.05.032 18639219 10.1016/j.ajo.2008.05.032

[14] Hirata M, Tsujikawa A, Matsumoto A, Hangai M, Ooto S, Yamashiro K, et al Macular choroidal thickness and volume in normal subjects measured by swept-source optical coherence tomography. Invest Ophthalmol Vis Sci. 2011;52:.4971–4978. doi: 10.1167/iovs.11-7729 21622704 10.1167/iovs.11-7729

[15] Copete S, Flores-Moreno I, Montero JA, Duker JS, Ruiz-Moreno JM. Direct comparison of spectral-domain and swept-source OCT in the measurement of choroidal thickness in normal eyes. Br J Ophthalmol. 2014;98: 334–338. doi: 10.1136/bjophthalmol-2013-303904 24288394 10.1136/bjophthalmol-2013-303904

[16] Agawa T, Miura M, Ikuno Y, Makita S, Fabritius T, Iwasaki T, et al Choroidal thickness measurement in healthy Japanese subjects by three-dimensional high-penetration optical coherence tomography. Graefes Arch Clin Exp Ophthalmol. 2011 10;249:10: 1485–1492. doi: 10.1007/s00417-011-1708-7 21556938 10.1007/s00417-011-1708-7

[17] Chhablani J, Rao PS, Venkata A, Rao HL, Rao BS, Kumar U, et al Choroidal thickness profile in healthy Indian subjects. Indian J Ophthalmol. 2014;62: 1060–1063. doi: 10.4103/0301-4738.146711 25494246 10.4103/0301-4738.146711 PMC4290194

[18] Li XQ, Larsen M, Munch IC. Subfoveal choroidal thickness in relation to sex and axial length in 93 Danish university students. Invest Ophthalmol Vis Sci. 2011;52: 8438–8441. doi: 10.1167/iovs.11-8108 21917938 10.1167/iovs.11-8108

[19] Wei WB, Xu L, Jonas JB, Shao L, Du KF, Wang S, et al Subfoveal choroidal thickness: the Beijing Eye Study. Ophthalmology 2013;120: 175–180. doi: 10.1016/j.ophtha.2012.07.048 23009895 10.1016/j.ophtha.2012.07.048

[20] Ikuno Y, Kawaguchi K, Nouchi T, Yasuno Y. Choroidal thickness in healthy Japanese subjects. Invest Ophthalmol Vis Sci. 2010;51: 2173–2176. doi: 10.1167/iovs.09-4383 19892874 10.1167/iovs.09-4383

[21] Ding X, Li J, Zeng J, Ma W, Liu R, Li T, et al Choroidal thickness in healthy Chinese subjects. Invest Ophthalmol Vis Sci. 2011;52: 9555–9560. doi: 10.1167/iovs.11-8076 22058342 10.1167/iovs.11-8076

[22] Margolis R, Spaide RF. A pilot study of enhanced depth imaging optical coherence tomography of the choroid in normal eyes. Am J Ophthalmol. 2009;147: 811–815. doi: 10.1016/j.ajo.2008.12.008 19232559 10.1016/j.ajo.2008.12.008

[23] Ruiz-Medrano J, Flores-Moreno I, Peña-García P, Montero JA, Duker JS, Ruiz-Moreno JM. Macular choroidal thickness profile in a healthy population measured by swept-source optical coherence tomography. Invest Ophthalmol Vis Sci. 2014;55:.3532–3542. doi: 10.1167/iovs.14-13868 24845638 10.1167/iovs.14-13868

[24] Ruiz-Moreno JM, Flores-Moreno I, Lugo F, Ruiz-Medrano J, Montero JA, Akiba M. Macular choroidal thickness in normal pediatric population measured by swept-source optical coherence tomography. Invest Ophthalmol Vis Sci. 2013;54: 353–359. doi: 10.1167/iovs.12-10863 23249703 10.1167/iovs.12-10863

