# Supplementary material for: Choroidal thickness in normal Indian subjects using Swept source optical coherence tomography
Source: PLoS One. 2018 May 16;13(5):e0197457. doi: 10.1371/journal.pone.0197457 (PMC5955571; doi:10.1371/journal.pone.0197457)
Supplement: S1 Table — (DOCX) [file pone.0197457.s001.docx]

Supplementary Table 1. Statistical tests used in the study

| S No. | Analysis | Statistical tests |
| --- | --- | --- |
| 1 | Comparison of systemic parameters between different age groups | Kruskal wallis, post hoc Mann Whitney |
| 2 | Comparison of CT between different age groups | One Way ANOVA post hoc Bonferoni |
| 3 | Comparison of CT between quadrants for each age group | One Way ANOVA post hoc Bonferoni |
| 4 | Comparison of choroidal thickness between male and female | Independent t test |
| 5 | Comparison of demographics between male and female | Mann Whitney U test & Independent t test |
| 6 | Effect of age on choroidal thickness | Spearman’s correlation |
| 7 | Effect of refractive error on choroidal thickness | Spearman’s correlation |
| 8 | Effect of axial length on choroidal thickness | Pearson correlation |
| 9 | Effect of ocular perfusion pressure on choroidal thickness | Pearson correlation |
| 10 | Prediction of choroidal thickness with associated factors | Linear regression |
| 11 | Intra observer repeatability in manual measurement of OCT | Intra class correlation coefficient |
| 12 | Inter Observer variation in manual measurement of OCT | Bland Altman Plot |

CT=Choroidal thickness; OCT=Optical coherence tomography
